# Pulmonary Functions and Health-Related Quality of Life among Silica-Exposed Workers

**Published:** 2017

**Authors:** Hamzeh Mohammadi, Somayeh Farhang Dehghan, Farideh Golbabaei, Saman Roshani, Reza Pakzad, Parvin Foroughi, Roohalah Hajizadeh

**Affiliations:** 1 Department of Occupational Health, School of Public Health, Tehran University of Medical Sciences, Tehran, Iran.; 2 Department of Occupational Health, School of Public Health, Shahid Beheshti University of Medical Sciences, Tehran, Iran.; 3 Department of Occupational Health, Tehran University of Medical Sciences, Tehran,Iran.; 4 Department of Occupational Health, Student Research Committee, Tehran University of Medical Sciences, Tehran, Iran.; 5 Department of Epidemiology and Biostatistics, School of Public Health, Tehran University of Medical Sciences, Tehran, Iran.; 6 Occupational Health Research Center, Qom University of Medical Sciences, Qom, Iran.

**Keywords:** Silica exposure, Lung function, Quality of life, Insulator manufacturer

## Abstract

**Background:**

This study aimed to investigate the pulmonary functions of silica-exposed workers and their health-related quality of life in an insulator manufacturing industry.

**Materials and Methods:**

In this cross-sectional study, participants selected from the manufacturing unit (n = 127) constituted the exposed group and those from the administrative department (n = 30) constituted the unexposed group. All subjects were evaluated using personal air sampling of crystalline silica, pulmonary function tests, and a quality of life questionnaire (36-item short form health survey [SF-36]).

**Results:**

The mean (SD) concentrations of crystalline silica were 0.507 (0.23) mg/m^3^ and 0.0116 (0.008) mg/m^3^ for the exposed and unexposed groups, respectively. All the pulmonary function indices and all the physical and mental health domains of the workers were significantly lower than those of the administrative clerks (p < 0.05). The silica concentration did not significantly correlate with the quality of life components and all the pulmonary function indices (p > 0.05), except for forced vital capacity (FVC) and forced expiratory volume in one second (FEV1) (p < 0.05).

**Conclusion:**

In conclusion, the workers exposed to higher levels of crystalline silica had lower values of pulmonary function indices and lower health-related quality of life; however, further follow-up studies are needed to confirm these findings.

## INTRODUCTION

Occupational respiratory diseases rank among the most common occupational diseases, and are usually latent for a long time; hence, the disease is diagnosed during the final stages, when therapeutic measures are usually ineffective (1). Several acute and chronic pulmonary diseases are caused by inhaling hazardous chemical agents at the workplace, including dust and toxic particles, metal fumes, gases and vapors, and other air-borne pollutants (2).

Among them, the mineral dust contains various compounds and can cause various pulmonary diseases, depending on the individual affected (3,4). Silica is regarded as one of the most important minerals with different industrial uses, such as in the casting process, preparation of detergent powder, and grinding process, and as a raw material for manufacturing insulators, tiles, and ceramic (5,6).

The insulator manufacturing industry is associated with a risk of silica emission and its consequences. An insulator is a device that has a high electrical resistance and is used as a good electrical insulation between two conductors with different voltage levels; it may also be used between a conductor and the earth. Several countries, such as the United States, China, Spain, and Iran produce these silicon insulators and their employees are exposed to the air-borne pollution that results from milling, mixing, pressing, assembling, cutting, grinding, and extruding the equipment during its production process. Silicon is needed to strengthen the insulator substrate (7).

Silicosis is the most common occupational lung disease and is caused by continuous exposure to dust containing free silica. This can lead to complications, such as pulmonary infections, pulmonary tuberculosis, pneumothorax, cardiac failure, and hemoptysis (8). Besides silicosis, occupational silica exposure has also been linked to lung cancer and silica has been classified by the International Agency for Research on Cancer (IARC) as a carcinogenic agent for humans (9). In the ceramic and pottery industries of Italy, a significant reduction in the lung function parameters, including the forced vital capacity (FVC) and forced expiratory volume in the first second (FEV1) have been reported (10). Gholamie et al. reported that respiratory complaints in the silica-exposed group were significantly higher than in the unexposed group; moreover, the spirometry indices of the exposed group, except for FEV1/FVC, were significantly lower than those of the unexposed group (11).

Health-related quality of life is a multidimensional concept that includes the functional ability and mental health of a person; it is influenced by various factors, including physical/mental status, economic circumstances, personal beliefs, and individual-environment interactions (12). Occupation is a key factor affecting the health-related quality of life (13). Occupational environments consist of physical, mental, and social stimuli, each of which may be regarded as a stress factor (14). These stresses have detrimental effects on both physical (its health and function) and mental health (15). Considering the poor work conditions in the factories of developing countries, the routine life of workers may be adversely affected by environmental factors in various aspects, such as physical, mental, social, and economic. Hence, the concept of health-related quality of life becomes significant in these people (16).

The effects of occupational silica exposure on the pulmonary function have been reported in many studies (17); however, health problems, such as respiratory impairments, may be associated with a worse quality of life, and there are few studies that consider it in silica-exposed workers. Silicosis is an irreversible disease and is caused by exposure to free silica. Silicosis can lead to a reduction in the lung function of the exposed workers and interferes with their activities of daily living and work (18). Consequently, it leads to a decline in their quality of life; however, there are few studies that evaluate the effect of the silica exposure and lung function on the quality of life of the workers (18). Therefore, the present study aimed to investigate the pulmonary functions and the health-related quality of life of silica-exposed workers in an insulator manufacturing industry, to help in developing programs that enhance the level of health in the workers of this industry.

## MATERIALS AND METHODS

### Participants

This cross-sectional study was conducted among the workers in the manufacturing workshops (the exposed group) and the administrative department (the unexposed group) in one of the insulator factories in Saveh, Iran. Since the number of unexposed subjects was lower than the exposed subjects, to enhance the power of the study, the ratio of exposed to unexposed participants was considered to be 4. This ratio corresponded to the maximum power achievable by the study. Assuming α = 0.05, 1 - β = 0.80, and the effect size = 0.50, the sample size was calculated to be 157, in which 127 subjects belonged to the exposed group and 30 subjects to the unexposed group. G*power software (version 3.1.9.2; by Franz Faul, Uni Kiel, Germany) was used for the sample size calculation (19,20). The participants were selected using a simple random method from the employees who are non-smokers and had been employed for at least five years and did not have any respiratory diseases, such as sinusitis, asthma, infection of the respiratory system, chronic bronchitis, chronic obstructive pulmonary disease, pneumoconiosis, or pulmonary fibrosis, reported in their medical records. A signed informed consent was obtained from all participants; they were required to fill out a demographic questionnaire and a questionnaire on respiratory symptoms over the past year, including cough, phlegm, wheeze, and dyspnea. Ethical approval was granted by the Research Ethics Committee of Tehran University of Medical Sciences.

### Measurement of silica concentration

Air sampling of crystalline silica was performed according to Method 7601 (21) of the National Institute for Occupational and Safety (NIOSH), using a polyvinylchloride (PVC) membrane filter (25 mm, 0.5 μm, SKC Inc., USA), nylon cyclone and a personal sampling pump with a flow rate of 1.7 L/min (224-PCMTX8, SKC Inc., USA). The crystalline silica was analyzed based on the NIOSH Method 7601, using a visible absorption spectrophotometer (DU-800 UV/Vis; Beckman Coulter, Inc.; United States).

### Pulmonary function tests

The pulmonary function tests were performed to evaluate the capacity and volume of the lungs among the participants. Spirometry measurements, including FVC, FEV1, ratio of FEV1 to FVC (FEV1/FVC), forced expiratory flow 25–75% (FEF 25–75%), and peak expiratory flow (PEF), were obtained before the work shift. A Minispir spirometer (S/N T02123, Italy) was used for the spirometry tests by an occupational medicine specialist. The spirometer was calibrated using a 1-L standard syringe each day, according to the instructions. The average percentage predicted for each of the lung function parameters was calculated and estimated based on the age, height, and gender by the spirometer. The participants were requested to discontinue drugs that might affect the respiratory system 24 hours before the test. In addition, all participants were trained before the spirometry test (10, 22). The spirometry data were analyzed by comparing the obtained values to the predicted values. For a normal spirometry, the FVC and FEV1 should be equal to or greater than 80% of the predicted value, and the FEV1-to-FVC ratio should be no more than 8–9 absolute percentage points below the predicted ratio (23).

### Health-related quality of life assessment

The assessment of health-related quality of life was performed using the 36-item self-report short form questionnaire (SF-36). It measured eight multi-item domains, including physical functioning (PF), social functioning (SF), role limitation due to physical health (RP), role limitation due to emotional problems (RE), emotional well-being or mental health (MH), energy/fatigue or vitality (VT), pain or bodily pain (BP), and general health (GH) (24). Each item was scored from 0 (worst possible health status) to 100 (best possible health status) (25) There were two general factors within the SF-36 that functioned as complementary descriptors of the overall health: the Mental Component Summary (MCS) and Physical Component Summary (PCS). Using these two components, general health examination was conducted with using fewer items and the results interpreted. Physical measures include items PF, RP, BP, GH, and the mental measure, which included items MH, RE, SF, and VT (26). The validity and reliability of the Iranian version of the questionnaire were approved by a previous study (27).

### Statistical analysis

The SPSS statistical software (version 20, SPSS Inc, Chicago, IL) was used for all statistical analyses. Descriptive (mean and standard deviation) and analytical (Student’s t-test, Pearson correlation coefficient, Spearman correlation coefficient, Mann-Whitney U test, and chi-square test) statistics were used. The Kolmogorov-Smirnov statistical test was performed for checking the normality of the obtained data. The significance level was set at *p* < 0.05.

## RESULTS

Table 1 presents the comparison of the mean (SD) demographic characteristics between the exposed and unexposed groups. Of the 157 subjects who participated in this survey, 127 were selected from the manufacturing unit (the exposed group) and 30 from the administrative department (the unexposed group); the mean (SD) ages were 39.28 (4.51) years and 39.03 (8.6) years for the exposed and unexposed groups, respectively. In the statistical analyses, there were no significant differences between the two groups in terms of demographic characteristics, including age, duration of employment, height, and weight (P>0.05; Student’s t-test).

**Table 1.** Demographic data of the study population

**Characteristic**	**Mean (SD)**		**p-value**

	**Exposed (*n* = 127)**	**Unexposed (*n* = 30)**	
**Age (years)**	39.28 (4.51)	39.03 (8.61)	0.871
**Duration of employment (years)**	11.92 (2.83)	15.11 (6.71)	0.171
**Height(cm)**	174.06 (6.28)	173.13 (7.11)	0.479
**Weight(kg)**	78.04 (11.03)	81.00 (19.44)	0.651

The prevalence rates of all respiratory symptoms were higher in the exposed group, compared to the unexposed group; however, dyspnea and cough alone showed statistical significance (*p* < 0.001; chi-square test).

The mean (SD) crystalline silica concentrations were 0.507 (0.23) mg/m^3^ and 0.0116 (0.008) mg/m^3^ for the exposed and unexposed groups, respectively. The mean difference between the two groups was statistically significant (*p* < 0.001); moreover, the workers of the manufacturing unit were exposed to concentration excursions higher than the threshold-limit value - time-weighted average (TLV-TWA = 0.025 mg/m^3^) recommended by the American Conference of Governmental Industrial Hygienists (ACGIH) (28).

The results of spirometric testing (Table 2) showed that there were significant differences between the spirometry parameters of the two groups (*p* < 0.05); all the pulmonary function indices of the workers were significantly lower than those of the administrative clerks.

**Table 2.** Values of pulmonary function indices

**Pulmonary function indices**	**Mean (SD)**		***P-value***

	**Exposed**	**Unexposed**	
**FVC (% pred)**	87.76 (7.96)	92.14 (9.81)	0.011
**FEV1 (% pred)**	85.45 (11.88)	93.84 (12.12)	0.001
**FEV1.FVC (%)**	97.45 (11.06)	102.71 (14.84)	0.03
**FEF25–75 (% pred)**	88.64 (16.31)	95.07 (7.91)	0.002
**PEF (L/S)**	99.84 (9.11)	105.36 (10.36)	0.004

There were weak but significant positive correlations between the ages of all participants and the pulmonary functions, including FVC, FEV1/FVC, and FEF 25–75% (*p*<0.03; r=0.34; Pearson correlation test); weak insignificant negative relationships were observed for FEV1 and PEF (*p*>0.05; r=−0.10). The relationships between the duration of employment for the exposed group and all pulmonary functions were statistically significant (*p*<0.03), except for FEV1/FVC (*p*>0.05); moreover, weak positive correlations (r=0.42) were present for all of them. The percentages of exposed and unexposed subjects with abnormal indices (FVC and FEV1 were both less than 80%) were 33.07 and 23.33, respectively.

The results of the Kolmogorov- Smirnov (KS) test showed that none of the psychosocial variables followed a normal distribution (p<0.05), except for two components: physical and mental health (p>0.05). The results obtained from the SF-36 (Table 3) demonstrated that all the physical and mental health variables for the exposed group were significantly lower than those for the unexposed group (*p* <0.05; Mann-Whitney U test).

**Table 3.** Scores for health-related quality of life questionnaire

**Variable**	**Exposed**	**Unexposed**	***P***
**Physical function**	83.62 (19.85)	94.5 (12.54)	0.001
**Rule limitation due to physical health**	80.51 (19.89)	89.16 (18.19)	0.023
**Rule limitation due to emotional problems**	79.79 (23.81)	88.88 (20.21)	0.044
**Energy/fatigue**	70.86 (13.69)	76.33 (9.27)	>0.001
**Emotional well-being**	74.85 (12.28)	81.73 (10.54)	0.002
**Social function**	73.72 (13.98)	80.01 (16.61)	0.009
**Pain**	77.36 (21.33)	87.01 (15.92)	0.024
**General health**	70.74 (15.36)	79.33 (10.56)	0.003

The correlation analysis of the quality of life and the ages of all participants showed that there were weak but significant positive relationships (p<0.04; r=0.32). Further, individuals within the age range of 30–40 years had the lowest scores for all domains of the quality of life in comparison with those in the age range of < 30 years and > 40 years.

Table 4 presents the relationships between the exposure to crystalline silica, pulmonary function indices, and two main components of the health-related quality of life, including physical and mental health. As can be inferred from Table 4, the silica concentration significantly correlated with the two components of the quality of life and all the lung function tests (*p* > 0.05), except for FVC and FEV1 (*p* < 0.015). Moreover, significant relationships were not present between all the pulmonary function indices and the two components of the health-related quality of life (*p* > 0.05; Spearman test). In addition, these correlations were relatively weak.

**Table 4.** Relationship between workers’ exposure to crystalline silica and pulmonary function indices and two main components of health-related quality of life

**Variable**	**Physical health component**	**Mental health component**	**Silica concentration (mg/m^3^)**
**Silica Concentration (mg/m^3^)**	r=−0.21	r=−0.342	-
	*p*=0.266	*p*=0.834	
**FVC (% pred)**	r=0.209	r=0.101	r=−0.368
	*p*=0.268	*p*=0.595	*p*=0.012
**FEV1 (% pred)**	r=0.791	r=0.131	r=−0.387
	*p*=0.678	*p*=0.945	*p*=0.014
**FEV1.FVC (%)**	r=−0.226	r=0.215	r=0.082
	*p*=0.231	*p*=0.254	*p*=0.221
**FEF25–75 (% pred)**	r=−0.018	r=−0.102	r=0.059
	*p*=0.533	*p*=0.592	*p*=0.443
**PEF (L/S)**	r=0.144	r=0.369	r=0.023
	*p*=0.448	*p*=0.055	*p*=0.630

## DISCUSSION

In the present study, the spirometric indices and health-related quality of life were compared between the workers who were exposed to silica dust and the employees of the administrative department in an insulator manufacturing industry. The results of air monitoring suggested that the levels of crystalline silica emission from the process units were approximately 20 times higher than the threshold-limit value - time-weighted average. These findings imply that there might be an increased risk of respiratory disorders and health problems for workers in response to silica dust exposure. The mean spirometric indices of the group exposed to high levels of silica dust were statistically significantly lower than those of the unexposed group. In addition, the exposed workers showed a higher prevalence of respiratory symptoms. Sakar et al. found that the workers of ceramic factories, who were exposed to silica dust, had higher pulmonary signs, including cough, mucus, and shortness of breath, in comparison to the non-exposed ones (29). Aminian et al. reported that the mean percentage of the spirometric indices, including FEV1/FVC, FVC and PEF, FEF 25–75%, and FEV1 in workers exposed to cement dust showed a significant reduction (22). Moreover, Hertzberg et al. investigated the respiratory symptoms and functional status of workers exposed to silica and found a decreased percentage-predicted FVC and FEV1 (30). The results of the present work agree with most findings of the above-mentioned studies.

Low but significant positive correlations were found between the duration of employment and all pulmonary functions in present study; Aminian et al. had indicated that an increase in the duration of employment could lead to a decrease in the pulmonary factors (31).

Since routine spirometry was used to test the lung function of the study subjects and it is well known that silica itself primarily affects the lung diffusion capacity. Further complementary studies may be performed using the diffusing capacity of the lung for carbon monoxide, besides spirometry (32). This may be important to detect early stage silica-related interstitial changes related to the reportedly high exposure levels.

No significant differences were observed between the two groups for the demographic variables; moreover, there were no air-borne contaminants with potentially damaging effects on the respiratory system and values higher than the TLV according to the reports of the health, safety, and environment sections of the factory studied; hence, conclusions on the potential reasons for the reductions in lung function parameters were evident.

Any health problem, especially pulmonary disorders that can cause a disruption in the normal life, can have a substantial impact on the quality of life (33). Based on the obtained results of the present research, the mean scores of all domains of the health-related quality of life in the exposed group were significantly lower than those of the unexposed group, and the lowest mean score in the exposed group was related to the dimension of general health. Stahl et al. found that all domains of the health-related quality of life could be significantly associated with the severity grades of the pulmonary disease, and the total scores of most of the mental and physical health domains were lower than those in patients with mild chronic obstructive pulmonary disease (34). Halvani et al. showed that 25.8% of the workers employed in lead and zinc mines did not have an acceptable standard of general health (35). The present study findings indicated that the office employees had higher scores and better health-related quality of life than did the manufacturing workers, which is in line with the findings of Salimzadeh et al. (36). Since the quality of life could be affected by several other parameters related to health, economic and environmental conditions; hence, it might be very difficult to make a definite decision and more comprehensive studies are needed to evaluate the possible effect of silica exposure on health-related quality of life.

Various studies have shown that age is a key factor in quality of life and increased age can result in a decreased quality of life, especially in the physical function and role-limitation due to physical health (37); however, the quality of life scores and the ages of the participants in the present study had weak but significant positive relationships.

The major limitations of this study were the small size of the unexposed control group and the lack of diffusion lung capacity testing; moreover, there were different types of confounding factors for the lung function test indices and quality of life: the socio-economic status of the participants, level of education and income, job security, and work-related stress for the quality of life; and non-occupational exposure for the respiratory function. Hence, further comprehensive studies should consider and adjust for these confounding variables.

## CONCLUSION

In general, the results showed that the workers who were exposed to higher levels of crystalline silica had lower pulmonary function indices, higher prevalence rate of respiratory symptoms, and lower health-related quality of life. Finally, it is difficult to draw definite conclusions about silica exposure and its deteriorating effects, especially on the quality of life of the workers in the insulator manufacturing industry; further follow-up studies are warranted to confirm these findings.
